# Experience of copy number variation sequencing applied in spontaneous abortion

**DOI:** 10.1186/s12920-023-01699-1

**Published:** 2024-01-08

**Authors:** Yi-Fang Dai, Xiao-Qing Wu, Hai-Long Huang, Shu-Qiong He, Dan-Hua Guo, Ying Li, Na Lin, Liang-Pu Xu

**Affiliations:** 1 College of Clinical Medicine for Obstetrics & Gynecology and Pediatrics, Fujian Medical University, Fuzhou, 350001 Fujian China; 2 Medical Genetic Diagnosis and Therapy Center of Fujian Maternity and Child Health Hospital, Fujian Provincial Key Laboratory for Prenatal Diagnosis and Birth Defect, No.18 Daoshan Road, Fuzhou, 350001 Fujian China

**Keywords:** High-throughput nucleotide sequencing, Genetics, Chromosome aberrations

## Abstract

**Purpose:**

We evaluated the value of copy number variation sequencing (CNV-seq) and quantitative fluorescence (QF)-PCR for analyzing chromosomal abnormalities (CA) in spontaneous abortion specimens.

**Methods:**

A total of 650 products of conception (POCs) were collected from spontaneous abortion between April 2018 and May 2020. CNV-seq and QF-PCR were performed to determine the characteristics and frequencies of copy number variants (CNVs) with clinical significance. The clinical features of the patients were recorded.

**Results:**

Clinically significant chromosomal abnormalities were identified in 355 (54.6%) POCs, of which 217 (33.4%) were autosomal trisomies, 42(6.5%) were chromosomal monosomies and 40 (6.2%) were pathogenic CNVs (pCNVs). Chromosomal trisomy occurs mainly on chromosomes 15, 16, 18, 21and 22. Monosomy X was not associated with the maternal or gestational age. The frequency of chromosomal abnormalities in miscarriages from women with a normal live birth history was 55.3%; it was 54.4% from women without a normal live birth history (*P* > 0.05). There were no significant differences among women without, with 1, and with ≥ 2 previous miscarriages regarding the rate of chromosomal abnormalities (*P* > 0.05); CNVs were less frequently detected in women with advanced maternal age than in women aged ≤ 29 and 30–34 years (*P* < 0.05).

**Conclusion:**

Chromosomal abnormalities are the most common cause of pregnancy loss, and maternal and gestational ages are strongly associated with fetal autosomal trisomy aberrations. Embryo chromosomal examination is recommended regardless of the gestational age, modes of conception or previous abortion status.

## Background

 Conventional G-banding karyotype analysis is widely used for the genetic analysis of miscarriage samples. However, this method is limited by low resolution, culture failure, poor chromosome morphology, long turnaround time, maternal cell contamination (MCC), and submicroscopic chromosomal variations that are not visible, all of which may lead to false negative results. Other methods such as fluorescence in situ hybridization and multiplex ligation-dependent probe amplification have also been used to identify the genetic causes of miscarriage [1–3]. However, none of these methods detect chromosomal abnormalities at the whole-genome level. Chromosomal microarray analysis (CMA) is a powerful technology for genetic diagnosis that can detect aneuploidy, submicroscopic chromosomal variations and so on at the genome-wide level. Nonetheless, a major shortcoming of CMA is its high cost, which restricts its use as a routine detection method for spontaneous abortions [4, 5].

Low-coverage (or low-pass) whole-genome next-generation sequencing (NGS) is a low-cost technique with a short turnaround time, unprecedented resolution, reliable high-throughput, and minimal DNA requirements. It has been widely used in clinics [6]. Compared to CMA, NGS has significant advantages in terms of quality, speed, and affordability [7–9]. Copy number variation sequencing (CNV-seq), an NGS-based method, has been used in most pediatric and prenatal diagnostic applications as a viable alternative to CMA because of its ability to simultaneously detect aneuploidies and submicroscopic chromosomal imbalances [9–11]. However, CNV-seq fails to detect MCC and polyploidy, limiting its application in abortion detection. Quantitative fluorescence polymerase chain reaction (QF-PCR) is a rapid chromosomal detection method commonly used in clinical settings. It can identify MCC, some euploidies such as triploid or tetraploid, and some common aneuploidies by amplification of selected short tandem repeat (STRs) sites and quantitatively analyzing allelic dosage ratios to evaluate the number of copies of specific chromosomes [12]. Therefore, we speculated that the combination of CNV-seq and QF-PCR would be a reliable approach for chromosome detection in POCs, as confirmed prenatally [11, 13].

Miscarriage is the spontaneous loss of a pregnancy at less than 28 weeks, or the spontaneous loss of the fetus with a weight less than 1000 g. When miscarriage occurs before 13 gestational weeks, it is called first- trimester miscarriage or early abortion; when it occurs from 13 to 28 gestational weeks it is called second-trimester miscarriage or late abortion [14]. Stillbirth is the death of a fetus in the uterus after 20 weeks of gestation [15]. The incidence of miscarriage is approximately 15–20%, with 25% of females experiencing at least one spontaneous abortion [16, 17]. Studies have shown that genetic factors play an important role in miscarriage, with 50% of cases caused by chromosomal abnormalities [18]. On the other hand, the risk factors for stillbirth (≥ 28 gestational weeks) are mainly immune and environmental factors [19]. Researchers have found that fetal chromosomal aneuploidy is the primary cause of miscarriage [20], with aneuploidy of chromosomes 13, 16, 18, 21, 22 and sex chromosomes being ubiquitous [21, 22]. Previous studies have focused on populations with specific clinical factors such as early or recurrent spontaneous abortion, and there have been few cross-sectional comparative studies of populations with these different factors. In this study, we aimed to evaluate the combined application of CNV-seq and QF-PCR as a tool for the identification of chromosomal abnormalities. We investigate the frequency and type of chromosomal aberrations in the POCs of participants under different clinical conditions to provide evidence for clinical advice and genetic counseling.

## Materials and methods

A total of 650 fetal specimens, including 597 chorionic villi and 53 fetal muscle tissues, were obtained from female participants who had undergone spontaneous abortion between April 2018 and December 2020. The mean age of the patients was 31.29 years old (19–46 years), and the mean gestational age was 9.1 weeks (5–25 weeks). Clinical information including early miscarriage history, normal live birth history, and mode of conception was recorded. Maternal age was classified into the following four groups: ≤ 29, 30–34, 35–39, and ≥ 40 years. The number of previous early miscarriages was classified into four groups: 0, 1, 2 and ≥ 3. The normal live birth history was categorized as “0” and “≥ 1” groups. The modes of conception were categorized as assisted and natural concepts.

This study was approved by the Protection of Human Ethics Committee of the Fujian Provincial Maternity and Children’s Hospital, which is affiliated with the Hospital of Fujian Medical University. Written informed consent was obtained from the individual or guardian participants.

### Copy number variation sequencing

CNV-seq was carried out in accordance with the manufacturer’s instructions. In brief, total genomic DNA was extracted from tissue samples using the Amp Genomic DNA Kit (TIANGEN Biotech, Beijing, China). After shearing the genomic DNA to an average size of 200 bp, 2.5 ng of the fragmented DNA was used to create the sequencing library. 8-bp bar-coded sequencing adaptors were legated to the DNA fragments, and PCR was performed to amplify the ligation products. The generated libraries were then pooled and sequenced on a NextSeq CN 500 high-throughput platform at approximately 1× depth after purification of the PCR product using magnetic beads. For each sample, 8–10 million of 35-bp single-end raw reads were produced. Short reads were aligned to the human reference genome (hg19) using the BWA aligner after sequencing quality control and trimming. Each reference chromosome was divided equally by a 100-kb window and the number of uniquely mapped reads in each window of each chromosome was counted. The LOWESS model was used to adjust the GC-bias of per window read counts. The corrected read counts were contrasted with an internal reference database created from a collection of 100 samples with a normal karyotype that was verified using G-banded karyotype analysis. A full description of the algorithms employed for the bioinformatics analysis was detailed in the previous literature [23]. Mosaicism was reported when the detection threshold of 10% was exceeded, CNVs detected by the platform had an effective minimum resolution of 100 kb.

### QF-PCR

Maternal peripheral blood samples were obtained via QF-PCR. DNA was extracted from maternal blood and POCs using a QIAGEN kit (Qiagen, Hilden, Germany), according to the manufacturer’s instructions. Multiple QF-PCRs were performed using a Chromosome (13/18/21/X/Y) multiplex STR Genotyping Kit (Guangzhou Darui Biotechnology Co., Ltd.) containing 20 STR markers (14 STR markers on autosomes 13, 18, and 21, four on chromosome X-linked markers, one on amelogenin, and SRY on chromosome Y). PCR products were separated on an ABI 3500 (Applied Biosystems, Foster City, CA, USA) capillary genetic analyzer and the results were analyzed using ABI Genemapper 6.0. The informative markers present in the POC DNA samples were compared with those in the maternal DNA samples to estimate the presence of maternal cell contamination.

### Evaluation of CNVs

Databases (ISCA, DGV, Decipher, Ensemble, OMIM, ClinGen, UCSC and PubMed) were used to analyze the suspected pathogenic regions. Pathogenicity of CNVs was evaluated according to the American College of Medical Genetics (ACMG) guidelines [24, 25]. CNVs were classified into three major categories: pathogenic, variants of uncertain significance (VOUS), and benign. Only pathogenic CNVs and VOUS were reported in this study.

### Statistical analysis

SPSS software (version 22.0) was used for the data analysis. Quantitative data were expressed as mean ± standard deviation (X ± S), and comparisons between groups were performed using the t-test. Qualitative data were represented as the number of cases (percentage), and comparisons between groups were performed using the paired chi-square test. Logistic regression analysis was used to analyze the factors related to chromosomal abnormalities. Differences were considered statistically significant at *P* < 0.05.

## Results

CNV-seq and QF-PCR were used to analyze 650 samples of aborted tissues collected during early and middle pregnancy. The success rate of all the tests was 100%. The rate of chromosomal abnormalities was 54.6% (355/650), of which 37.1% (241/650) were single aneuploidies, 2.8% (18/650) were multiple aneuploidies, 5.2% (34/650) were polyploidy, 3.5% (23/650) were mosaic aneuploidies, and 6.2% (40/650) were pathogenic copy number variations (pCNVs). VOUS were identified in 60 cases (9.2%), and normal results were identified in 235 cases (36.2%). Most aneuploidies were autosomal trisomies (217/650, 33.4%), whereas the others were monosomies found on chromosomes X (39/241, 16.2%) and 21 (3/241, 1.2%) (Table 1).

**Table 1:** Baseline characteristics and details of the 650 cases of chromosomal abnormalities

Characteristics	Number	Proportion (%)
Age of mother who had miscarriages (31.29 ± 4.55 years)		
≤ 29	255	39.2
30–34	234	36.0
35–39	128	19.7
≥ 40	33	5.1
Gestational week of fetuses(9.1 ± 2.42 weeks)		
Early pregnancy(≤ 12 weeks)	597	91.8
Middle pregnancy(13–28 weeks)	53	8.2
Aneuploidy	259	39.9
Autosomal trisomy	217	33.4
Monosomy X	39	6.0
Autosomal monosomy	3	0.45
Sex chromosome trisomy	2	0.3
Mosaicism	23	3.5
Polyploidy	34	5.2
pCNV	40	6.2
VOUS CNV	60	9.2
Normal	235	36.2

**Fig. 1:**
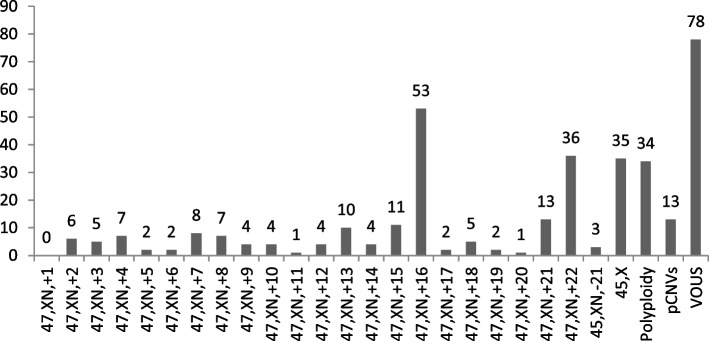
Distribution of chromosomal abnormalities in early abortion

**Fig. 2:**
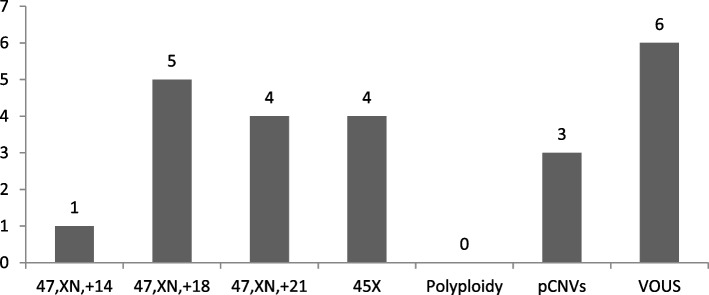
Distribution of chromosomal abnormalities in late abortion

Different distributions of chromosomal abnormalities were detected between first- and second-trimester abortions. In the first trimester of pregnancy loss, all chromosomes were involved in trisomies (except for chromosome 1), with T16 being the most common finding, followed by T22, T21, T18, and T15. Monosomy X was the most frequently encountered sex chromosome abnormality, with an incidence of 6.0% (Fig. 1). Seventeen abnormalities occurred in second-trimester miscarriages, and these abnormalities mainly involved T18, T21, 45, X and pCNVs. The most frequent karyotype was trisomy 18 (29.4%, 5/17), followed by monosomy X (23.5%, 4/17), trisomy 21 (23.5%, 4/17), and pCNVs (17.6%, 3/17) (Fig. 2).

Associations between chromosomal abnormalities and gestational age, maternal age, previous miscarriages, live birth history, and mode of conception are shown in Table 2. The rate of chromosomal abnormalities in the first-trimester pregnancy loss (56.6%) was significantly higher than that in the second-trimester pregnancy loss (32.1%) (*P* < 0.05). Autosomal trisomy was less common in second-trimester pregnancy loss than in first-trimester pregnancy loss (*P* < 0.05), but no statistical difference was found in the frequency of 45, X (Table 3). Similar incidences of chromosomal abnormalities were found among women aged ≤ 29, 30–34, and 35–39 years (*P* > 0.05), and were all significantly lower than those in women ≥ 40 years (*P* < 0.05). The incidence of autosomal trisomy also increased with maternal age (*P* < 0.05). The frequency of 45, X decreased with maternal age; however, the difference was not statistically significant (*P* > 0.05). The frequency of chromosomal abnormalities in miscarriages from women with a normal live birth history was 55.3%, it was 54.4% from women without a normal live birth history (*P* > 0.05), indicating that there was no significant difference in the frequency of CA between women with and without a normal live birth history. There were no significant differences in the rate of chromosomal abnormalities among miscarriages from women without, with 1, and with ≥ 2 previous miscarriages (*P* > 0.05). No significant differences were observed between miscarriages from women with different modes of conception (*P* > 0.05).

**Table 2:** Association between clinical information and the frequency of chromosomal abnormalities

	Normal n (%)	Abnormal n (%)	X2	*P*
Maternal age (years) (*N* = 650)			9.366	0.025
≤ 29 (*N* = 255)	105(41.2)	150(58.8)		
30–34 (*N* = 234)	83(35.5)	151(64.5)		
35–39 (*N* = 125)	41(32.8)	84(67.2)		
≥ 40 (*N* = 36)	6(16.7)	30(83.3)		
Previous miscarriage (*N* = 650)			2.051	0.562
0 time (*N* = 198)	137(46.0)	161(54.0)		
1 times (*N* = 206)	91(44.2)	115(55.8)		
2 times (*N* = 104)	44(42.3)	60(57.7)		
≥ 3 times (*N* = 42)	23(54.8)	19(45.2)		
Gestational age (*N* = 650)			11.828	0.001
first- trimester (*N* = 597)	259(43.4)	338(56.6)		
second- trimester (*N *= 53)	36(67.9)	17(32.1)		
Normal live birth history (*N* = 650)			0.045	0.831
No(*N* = 491)	224(45.6)	267(54.4)		
Yes(*N* = 159)	71(44.7)	88(55.3)		
Mode of conception (*N* = 650)			0.437	0.509
Natural conception (*N* = 568)	255(44.9)	313(55.1)		
Assisted conception (*N* = 82)	40(48.8)	42(51.2)

**Table 3:** Distribution profile and frequency of chromosomal abnormalities in different maternal and gestational ages

	CNVs (n, %)	Monosomy X (n, %)	Autosomal trisomy (n, %)
Gestational age			
< 13 weeks (*N* = 597)	91 (15.2)	35 (5.9)	204 (34.2)
≥ 13 weeks (*N* = 53)	9 (17.0)	4 (7.5)	10 (18.9)
X^2^	0.113	0.245	5.162
*P*	0.737	0.621	0.023
Maternal age			
≤ 29 (*N* = 255)	45 (17.6)	20 (7.8)	59 (23.1)
30–34 (*N* = 234)	43 (18.4)	12 (5.1)	75 (32.1)
35–39 (*N* = 125)	8 (6.4)	6 (4.8)	58 (46.4)
≥ 40 (*N* = 36)	4 (11.1)	1 (2.8)	22 (61.1)
X^2^	10.868	2.833	34.371
*P*	0.012	0.418	0.000

To identify significant CNVs related to miscarriage, cases with numerical chromosomal abnormalities were excluded from CNV analysis. As a result, a total of 60 pCNVs in 40 cases were subjected to further analysis, including 29 with duplications in 28cases and 31 with deletions in 29cases. The pCNVs of deletions and duplications ranged in size from 450 Kb–35.6 Mb and 0.38 Mb–217.86 Mb, respectively. The distribution of all detected pCNVs on all chromosomes was shown in Table 4. Deletions occurred mostly on chromosome 4, followed by chromosomes 8 and X. Duplications occurred mostly on chromosome 16. CNVs were less frequently detected in women with advanced maternal age than in women aged ≤ 29 years and 30–34 years (*P* < 0.05). However, no statistically significant differences were found in the frequency of CNVs at different gestational ages (*P* > 0.05). The results of the logistic regression analysis identified a trend suggesting that the percentage of fetal chromosomal abnormalities was significantly higher in advanced maternal age (OR = 1.810, 95% CI 1.217-2.693), and lower gestational age (OR = 0.361, 95% CI: 0.196–0.665) (Table 5).

**Table 4:** Details of 40 cases with pCNVs

Case	Maternal age	CNV-seq Results	Size range	Pathogenicity category	Associated syndrome
1	28	seq[hg19]dup(17)(p13.3)	0.38Mb	pathogenic	SHFLD3 syndrome
2	29	seq[hg19]dup(16)(p13.3)	1.65Mb	pathogenic	
3	24	seq[hg19]del(X)(p22.13)	1.34Mb	pathogenic	Epileptic encephalopathy, early infantile,
4	30	seq[hg19]del(21)(q11.2q21.3)	16.0Mb	pathogenic	/
		seq[hg19]dup(20)(p12.1q13.33)	47.42Mb	pathogenic	/
5	29	seq[hg19]del(1)(q43q44)	5.76Mb	pathogenic	MENTAL RETARDATION, AUTOSOMAL DOMINANT 22
		seq[hg19]dup(2)(q35q37.3)	23.88Mb	pathogenic	Syndactyly, type 1, with or without craniosynostosis
6	31	seq[hg19]dup(2)(p25.3q14.2)	121.92Mb	pathogenic	/
		seq[hg19]dup(16)(p13.3p12.3)	17.30Mb	pathogenic	16p13.3 duplication syndrome
7	26	seq[hg19]del(8)(p23.3p21.2)	26.18Mb	pathogenic	8p23.1 deletion syndrome
		seq[hg19]dup(4)(q28.1q35.2)	62.90Mb	pathogenic	/
8	29	seq[hg19]del(3)(q21.3q22.1)	2.96Mb	pathogenic	Immunodeficiency 21
9	32	seq[hg19]del(7)(q35q36.3)	12.38Mb	pathogenic	/
		seq[hg19]dup(7)(q32.3q35)	12.46Mb	pathogenic	/
10	25	seq[hg19]del(4)(q34.3q35.2)	13.02Mb	pathogenic	/
		seq[hg19]dup(9)(p24.3p13.1)	38.58Mb	pathogenic	/
11	29	seq[hg19]del(4)(p16.3p16.1)	5.98Mb	pathogenic	Wolf-Hirschhorn syndrome
12	25	seq[hg19]del(22)(q13.3)	1.5Mb	pathogenic	/
13	31	seq[hg19]dup(2)(p25.3q35)	217.86Mb	pathogenic	2q31.1 duplication syndrome
		seq[hg19]dup(8)(q24.11q24.3)	28.18Mb	pathogenic	/
14	29	seq[hg19]del(16)(p13.3)	1.64Mb	pathogenic	/
15	36	seq[hg19]dup(7)( q31.1-q31.33)	18.6Mb	pathogenic	/
16	29	seq[hg19]dup(18)(p11.32q23)	75.90Mb	pathogenic	/
17	28	seq[hg19]del(15)(q26.2q26.3)	4.62Mb	pathogenic	15q26-qter deletion syndrome
18	25	seq[hg19]del(8)(p23.3p23.2)	5.05Mb	pathogenic	/
19	27	seq[hg19]del(X)(p22.33)	0.88Mb	pathogenic	/
20	29	seq[hg19]del(4)(p16.3)	3.005Mb	pathogenic	Wolf-Hirschhorn syndrome
		seq[hg19]del(4)(p16.3p15.1)	25.22Mb	pathogenic	/
		seq[hg19]dup(4)(p15.1p13)	10.451Mb	VOUS	/
21	26	seq[hg19]del(Y)q11.1-q11.23)	15.68Mb	pathogenic	/
22	30	seq[hg19]dup(7)(q31.33q34)	15.225Mb	pathogenic	/
		seq[hg19]del(7)(q34q36.3)	17.5Mb	pathogenic	Kleefstra syndrome 2, Holoprosencephaly 3 Currarino syndrome
23	30	seq[hg19]del(5)(p15.33p14.3)	22.8Mb	pathogenic	Cri du Chat syndrome
24	31	seq[hg19]del(X)( p22.31)	1.80Mb	pathogenic	/
25	27	seq[hg19]del(8)(p23.3q11.1)	46.85Mb	pathogenic	Monosomy 8p syndrome
		seq[hg19]dup(8)(q11.1q24.3)	99.355Mb	pathogenic	Trisomy 8q syndrome
26	30	seq[hg19]dup(22)(q13.2q13.31),	750Kb	VOUS	/
		seq[hg19]del(22)(q13.31q13.33)	6.9Mb	pathogenic	Phelan-McDermid syndrome
27	31	seq[hg19]del(8)(p23.3p23.1)	6.8Mb	VOUS	/
		seq[hg19]dup(8)(p12p12)	1.45Mb	VOUS	/
		seq[hg19]del(8)(p23.1p12)	22.25Mb	pathogenic	/
		seq[hg19]dup(8)(p11.1q24.3)	102.455Mb	pathogenic	Trisomy 8q syndrome
28	30	seq[hg19]dup(11)(q13.1q13.2)	1.8Mb	pathogenic	/
29	31	seq[hg19]dup(12)(q24.11q24.33)	24.35Mb	pathogenic	/
30	26	seq[hg19]dup(6)(p25.3q13)	74.95Mb	pathogenic	Trisomy 6p syndrome
31	25	seq[hg19]dup(16)( q21q24.3)	28.25Mb	pathogenic	/
		seq[hg19]del(10)( q21q24.3)	4.1Mb	pathogenic	/
32	27	seq[hg19]del(4)(p16.3p16.1)	9.124Mb	pathogenic	Wolf-Hirschhorn syndrome
		seq[hg19]dup(4)(p11q35.2)	141.37Mb	pathogenic	/
33	30	seq[hg9]del(X)(p21.1)	450Kb	pathogenic	/
34	32	seq[hg19]del(7)(q34q36.3)	49.775Mb	pathogenic	/
		seq[hg19]dup(7)(q21.2q34)	16.85Mb	pathogenic	Kleefstra syndrome 2 Holoprosencephaly 3 Currarino syndrome
35	28	seq[hg19]dup(16)(p11.2)	850Kb	pathogenic	16p11.2 duplication syndrome
36	25	seq[hg19]del(8)(p23.3p12)	35.6Mb	pathogenic	
37	28	seq[hg19]dup(5)(p15.33p15.2)	12.85Mb	pathogenic	Jacobsen syndrome
		seq[hg19]del(11)(q24.1q25)	14.95Mb	pathogenic	/
38	25	seq[hg19]del(4)(p16.3p15.32)	17.57Mb	pathogenic	Wolf-Hirshhorn syndrome
39	29	seq[hg19]dup(6)(q23.2q27)	39.75Mb	pathogenic	/
40	31	seq[hg19]dup(1)(p36.33p36.32)	1.65Mb	pathogenic	/
		seq[hg19]del(15)(q26.1q26.3)	8.25Mb	pathogenic	/

**Table 5:** Logistic regression analysis of chromosomal abnormalities in miscarriage samples

Variables	Regression coefficient	Standard error	WaldX2 value	*P* value	OR value	95%CI
Maternal age	0.594	0.203	8.579	0.003	1.810	1.217-2.693
Gestational age	-1.018	0.311	10.705	0.001	0.361	0.196-0.665

## Discussion

The overall detection rate of clinically significant chromosomal abnormalities was 54.6%. Additionally, the rate of VOUS was 9.2%, which is in accordance with previous studies [13, 26]. We found that the largest proportion of chromosomal abnormalities was autosomal trisomy (33.4%), followed by CNVs (15.4%), and monosomy (6.0%). The frequencies of aneuploidy and polyploidy (39.9% and 5.2%, respectively) in the present study were similar to those obtained in a large-scale study (42.5% and 7.5%, respectively) conducted by Sahoo et al. [13]. Trisomies T16 and T22 were the most common, followed by T21, T15, T18, and T13. Trisomy was detected on all chromosomes except T1.

The rate of chromosomal abnormalities in second-trimester miscarriages was as high as 32.1% in this study but was lower than that in early miscarriages (56.6%). The lower frequency of other chromosomal trisomies may be because most trisomic embryos end in embryo implantation failure, and not all embryos have the opportunity to manifest abortion after implantation. In contrast, fetus withT16, T22, and T15 routinely have no opportunity to survive; therefore, these fetuses are almost always miscarried in early pregnancy, implying that T16, T22, and T15 may affect embryo development more than implantation. The risk of chromosomal abnormalities was significantly lower in the mid-trimester group than in the early pregnancy group (26.4% vs. 50.4%, *P* < 0.05); however, it still had a high risk of occurrence during this period, and was the leading cause of embryonic abortion in the mid-trimester. Therefore, chromosomal testing is necessary to identify the cause of miscarriages, even in the second trimester. According to previous studies, 45,X, T21, and T18 are miscarried in early pregnancy, whereas some continue to develop and survive in mid and late pregnancy. Further scientific studies are needed to reveal the underlying mechanisms [27] that also support self-repair during further embryo development – including apoptosis and selective differentiation [28], resulting in a substantial decrease in the proportion of abnormal chromosomal mosaicism during mid-pregnancy. In our study, the incidence of polyploidy in early pregnancy was as high as 10.0% (34/338); no polyploidy was detected in the second-trimester, and 97.1% (33/34) were triploid.

Advanced maternal age (≥ 35 years ) is a well-known independent factor associated with the frequency of chromosomal abnormalities during miscarriages [29, 30]. In this study, the frequencies of chromosomal abnormalities in women aged up to 30 years and 30–34 years were similar, but lower than those in women aged 35–39 years; all of them were significantly lower than those in women aged ≥ 40 years. This tendency was consistent with that of autosomal trisomy, confirming a close association between maternal age and viable autosomal trisomy. In recent years, some studies have proposed that the incidence of post-meiotic abnormalities such as structural abnormalities is not directly related to maternal age [31, 32]. In our study, a higher frequency of aneuploidy and lower frequency of CNVs were identified in the advanced maternal age group. Our results further support the theory that the incidence of embryonic aneuploidy increases with maternal age. The frequent detection of CNVs in miscarriages from young women is interesting; however, the overall sample size was small, and CNV-seq was not applied for parents in this study. Parental analysis is very promising for future research, because it allowed to detect de novo and inherited CNV. The latter may have a considerable interest for couples with recurrent pregnancy loss. Monosomy X is the most common viable sex chromosome abnormality. Unlike viable autosomal trisomy, the frequency of monosomy X did not increase with maternal age, which agrees with previous reports [7, 29, 33]. Hassold et al. [33, 34] found that paternal sex chromosome loss was the most common error leading to 45, X. They speculated that monosomy X was more likely derived from a meiotic error of the father than the mother. There are two possible reasons for this: an increase in the frequency of monosomy X conceptions related to events in meiosis, fertilization, or early zygotic division; or an increase in the rate of survival of monosomy X conceptions to the stage of recognizable pregnancies.

Sub-microscopic genomic imbalances or CNVs have been shown to play an important role in prenatal ultrasound anomalies and neuron-developmental disorders such as intellectual disability, autism, and epilepsy [35, 36]. Several attempts have been made to identify lethal CNVs in humans. Analysis of the functions of the genes contained in the CNVs showed that the percentage of pathogenic CNV in miscarriage tissues ranged from 6 to 15% [32, 37, 38]. The detection rate of CNVs in our study was 15.4%, including 6.2% of pathogenic CNVs. Among these cases, 4p16.3 microdeletion, 8p23.3microdeletion, 16p13.3microdeletion, 16p13.3 duplications and 16q24.3 duplications were found, some of which have also been reported in other studies concerning miscarriage [39, 40]. These microdeletions and microduplications might be related to pregnancy loss by comparing the prevalence of CNVs in miscarriage products and the general population; however, there is still no definite conclusion owing to the lack of more powerful evidence. Therefore, large-scale studies are required to confirm whether these CNVs cause miscarriages.

The present study had some limitations. First, the overall sample size was small, particularly for the mid-trimester. More cases, especially those of mid-trimester miscarriages, should be collected in future studies, and further functional studies should be performed on CNVs and genes associated with miscarriage. Second, parental karyotyping was not offered to couples whose POC revealed pCNVs abnormalities. Third, the distribution of patients was unequal between the age groups.

## Conclusions

Our results confirmed that chromosomal abnormalities were the most common cause of pregnancy loss. Maternal and gestational ages are strongly associated with fetal chromosomal aberrations. Embryo chromosomal examination is recommended regardless of the gestational age, mode of conception or previous abortion status. Some useful and accurate genetic etiology information was obtained, which provides useful genetic guidance for high-risk pregnancies.

## Data Availability

All data relevant to the study are included in the article. The datasets generated during the current study are available in the SRA repository, https://www.ncbi.nlm.nih.gov/sra/PRJNA961959.

## Abbreviations

CNV-seq: Copy number variation sequencing
CA: Chromosomal abnormalities
POCs: Products of conception
pCNVs: Pathogenic copy number variants
MCC: Maternal cell contamination
CMA: Chromosomal microarray analysis
NGS: Next-generation sequencing
QF-PCR: Quantitative fluorescence polymerase chain reaction
STRs: Short tandem repeat
VOUS: Variants of uncertain significance
