# Correction: Experience of copy number variation sequencing applied in spontaneous abortion

**DOI:** 10.1186/s12920-025-02118-3

**Published:** 2025-03-10

**Authors:** Yi‑Fang Dai, Xiao‑Qing Wu, Hai‑Long Huang, Shu‑Qiong He, Dan‑Hua Guo, Ying Li, Na Lin, Liang‑Pu Xu

**Affiliations:** 1 College of Clinical Medicine for Obstetrics & Gynecology and Pediatrics, Fujian Medical University, Fuzhou, Fujian 350001 China; 2 Medical Genetic Diagnosis and Therapy Center of Fujian Maternity and Child Health Hospital, Fujian Provincial Key Laboratory for Prenatal Diagnosis and Birth Defect, No.18 Daoshan Road, Fuzhou, Fujian 350001 China

**Correction to: Dai et al. BMC Medical Genomics (2024) 17:15**

10.1186/s12920-023-01699-1

Following the publication of the original article, the authors reported the affiliations were erroneously written. Medical Genetic Diagnosis and Therapy Center of Fujian Maternity and Child Health Hospital should have been a part of Affiliation 2, instead of Affiliation 1. The affiliations in this correction article are correct.

The original article has been corrected.

